# Serum endocan levels in relation to traditional and non-traditional anthropometric indices in adult population

**DOI:** 10.5937/jomb0-25170

**Published:** 2021-01-26

**Authors:** Aleksandra Klisić, Nebojša Kavarić, Vesna Spasojević-Kalimanovska, Jelena Kotur-Stevuljević, Ana Ninić

**Affiliations:** 1 University of Montenegro, Faculty of Medicine, Primary Health Care Center, Center for Laboratory Diagnostics, Podgorica, Montenegro; 2 University of Montenegro, Faculty of Medicine, Primary Health Care Center, Podgorica, Montenegro; 3 University of Belgrade, Faculty of Pharmacy, Department for Medical Biochemistry, Belgrade

**Keywords:** cardiometabolic risk, endocan, inflammation, obesity, gojaznost, inflamacija, endokan, kardiometabolički rizik

## Abstract

**Background:**

Association between endocan and nontraditional anthropometric indices, as distinct cardiovascular disease risk factors, has not been examined in previous studies. Endocan is a novel inflammation biomarker with its higher levels involved in cardiometabolic diseases development. Taking into consideration that obesity is an independent risk factor for many cardiometabolic diseases, we aimed to explore the relationship between endocan levels and novel anthropometric indices [i.e., body adiposity index (BAI), cardiometabolic index (CMI), a body shape index, body roundness index, conicity index, lipid accumulation product index and visceral adiposity index] and traditional ones [i.e., waist circumference, hip circumference, body mass index, waist-to-height ratio and waist-to-hip ratio] in adult population.

**Methods:**

A total of 177 participants were included. Anthropometric indices and biochemical parametres were measured.

**Results:**

Univariate regression analysis demonstrated positive correlations of endocan and almost all anthropometric data. To explore independent associations of endocan and anthropometric parameters, the Model which fulfilled criteria for ordinal regression testing was created. Adjusted odds for BAI given in the Model (OR=1.120, 95% CI 1.036-1.212,* P*=0.004), demonstrated that a rise in BAI by 1 unit increased the probability of higher endocan concentration by 12%. As well, a rise in CMI for 1 unit, increased the probability for higher endocan levels for 2.6 times (OR=2.599, 95% CI 1.006-6.712, *P*=0.049). A total of 20.1% of variation in endocan levels could be explained by this Model.

**Conclusions:**

Non-traditional obesity indices, BAI and CMI independently correlated with higher serum endocan levels in adult population.

## Abstract (sr)

**Uvod:**

Povezanost endokana sa netradicionalnim antropometrijskim parametrima, kao različitim faktorima rizika za kardiovaskularne bolesti, nije ispitivana u prethodnim studijama. Endokan je novi biomarker inflamacije, čije su veće vrednosti zabeležene kod kardiometaboličkih poremećaja. Znajući da je gojaznost nezavisan faktor rizika za mnoga kardiometabolička oboljenja, cilj je bio da se ispita povezanost endokana i novih antropometrijskih pokazatelja [tj. indeks telesne gojaznosti (BAI), kardiometabolički indeks (CMI), indeks oblika tela, indeks zaokruženosti tela, indeks koniciteta, indeks produkata lipidne akumulacije i indeks visceralne gojaznosti] i onih tradicionalnih [tj. obim struka, obim kukova, indeks telesne mase, odnos obim struka/telesna visina, odnos obim struka/obim kukova] u populaciji odraslih.

**Metode:**

Ukupno 177 ispitanika je učestvovalo u istraživanju. Mereni su antropometrijski i biohemijski parametri.

**Rezultati:**

Univarijantna regresiona analiza je pokazala pozitivnu korelaciju endokana i skoro svih ispitivanih antropometrijskih parametara. U cilju daljeg ispitivanja postojanja nezavisnih korelacija endokana i antropometrijskih parametara, kreiran je Model koji je zadovoljio kriterijume za ordinalnu regresiju. Prilagođeni Odds-ovi za BAI u Modelu (OR=1,120, 95% CI 1,036-1,212, *P*=0,004), su pokazali da je porast BAI za 1 jedinicu povećao verovatnoću za porast koncentracije endokana za 12%. Takođe, porast CMI za 1 jedinicu, povećao je verovatnoću za veće vrednosti endokana za 2,6 puta (OR=2,599, 95% CI 1,006-6,712, *P*=0,049). Ukupno 20,1% varijabiliteta u vrednostima koncentracije endokana može biti objašnjeno ovim Modelom.

**Zaključak:**

Netradicionalni pokazatelji gojaznosti, BAI i CMI su nezavisno povezani sa većim vrednostima endokana u populaciji odraslih.

## Introduction

Endocan is an intriguing proteoglycan tightly connected with endothelial dysfunction and concomitant inflammation [1]. Recent studies have shown higher level of this inflammation marker in many cardiometabolic diseases, such as type 2 diabetes [2], chronic kidney disease [3], fatty liver disease [4], hypertension [5].

On the other hand, obesity is an established risk factor for such cardiometabolic diseases [6] [7]. The increased inflammation markers in adipocites and disbalance in free radicals production and antioxidant defence, increased flux of free fatty acids and dysfunction of hepatocytes, are some of the potential mechanisms linking obesity and cardiovascular disease (CVD). In addition to this, impaired insulin signaling pathways, diminished nitric oxide pro duction with associated vasoconstriction and endothelial dysfunction are also contributing mecha nisms to such pathophysiology [7] [8] [9] [10].

However, there are discordant results about relationship between serum endocan level and obesity. Namely, some studies reported lower endocan levels in the obese state [11], as well as an inverse association between circulating level of endocan and the most commonly used obesity indices, such as body mass index (BMI) [12] [13] [14] and waist circumference (WC) [15] in different population groups. Contrary to this, the others showed the positive association between circulating endocan level and mentioned obesity indices, BMI [16] [17] [18] and WC [18].

Knowing that several recent studies revealed discrepant results considering this relationship with traditional anthropometric parameters, we aimed to obtain deeper understanding into the potential link between obesity and serum endocan level. Importantly, no studies have examined the association between endocan and non-traditional anthropometric indices so far. In line with this, numerous researches suggest that novel non-traditional anthropometric parameters can be better indicators of higher cardio metabolic risk, than traditional commonly used ones [19] [20] [21] [22] [23] [24] [25].

Therefore, this study aimed to explore the relationship between endocan levels and new anthropometric indices [i.e., body adiposity index (BAI), cardiometabolic index (CMI), a body shape index (ABSI), body roundness index (BRI), conicity index (CI), lipid accumulation product index (LAP) and visceral adiposity index (VAI)] and traditional ones [i.e., BMI, WC, hip circumference (HC), waist-toheight ratio (WHtR) and waist-to-hip ratio (WHR)] in adult population.

## Materials and Methods

### Subjects

A total of 177 individuals (70.6% women) participated in this cross-sectional study. The research was conducted between May and July 2017 in the Primary Health Care Center in Podgorica, Monte negro. The Institutional Ethics Committee approved the research protocol. Participants were enrolled in a consecutive manner and each of them signed an informed consent of willingness to be included in the study.

The eligibility to enter the study were: subjects older than 18 years, free of diabetes, CVD, chronic kidney disease, carcinomas, autoimmune and inflammatory diseases, as well as without endocrine disorders (e.g., hypo- and hyperthyroidism, Cushing disease). Another criterion for the persons to be included in this study was that they were not under any weight loss programme and that their body weight did not vary in the previous 3 months.

Exclusion criteria were: alcohol consumption, acute inflammation [high sensitivity C-reactive protein (hsCRP) >10 mg/L], pregnancy, lactation, as well as biochemical confirmation of diabetes (i.e., fasting glucose ≥7.0 mmol/L, or glucose level ≥ 11.1 mmol/L, 2 hours after an oral glucose tolerance test, or glycated hemoglobin level ≥ 6.5% on two different determinations), anti-inflammatory and antidiabetic drugs use.

Traditional anthropometric parameters were taken (weight, height, WC, HC), and BMI, WHtR and WHR were calculated, as described elsewhere [19].

The BAI was calculated by the following equation [20]:

[HC (cm) ÷ height (m)^1.5^] – 18,

The BRI was calculated, as follows [21]:

BRI = 364.2−365.5 x √1−(WC/2π)^2^ / (0.5 x height)^2^,

The CI was calculated using the equation [22]:

CI = WC (cm) / [0.109 x weight (kg)/height (cm)]

ABSI was calculated, as follows [23]:

WC/(BMI^2/3^ x height^1/2^), expressed in m1^1/6^ kg^−2/3^.

The CMI was calculated using the formula [24]:

CMI = TG/HDL-c × WHtR,

where triglycerides (TG) and high density lipoprotein cholesterol (HDL-c) are expressed in mmol/L.

The VAI was calculated, as follows [25]:

{[WC/36.58 + (1.89 × BMI)] × (TG/0.81) × (1.52/HDL-c)} for women, and {[WC/39.68 + (1.88 × BMI)] × (TG/1.03) × (1.31/HDL-c)} for men,

where WC is expressed in cm, BMI in kg/m^2^, TG and HDL-c in mmol/L.

The LAP was calculated using the formula [26]:

LAP = [(WC - 58) x TG] for females, and [(WC-65) x TG] for males,

where WC is expressed in cm, and TG in mmol/L.

### Methods

The blood samples were taken in 7.00-9.00 a.m. after participants fasted at least 8 hours. In order to obtain sera, samples were left to clot within 30 minutes and then centrifuged at room temperature for 10 minutes at 3000xg.

Serum endocan levels were determined by using an enzyme-linked immunosorbent commercial assay (ab213776 – Human ESM1 ELISA Kit, Abcam, Cambridge, UK).

Immunoturbidimetric method was used for determination of HbA1c in samples of a whole blood [Roche Cobas c501 chemistry analyzer (Roche Diagnostics GmbH, Mannheim, Germany)]. Serum glucose and lipid parameters (i.e., TG and HDL-c) were measured spectrophotometrically, on the same autoanalyzer as HbA1c. Serum hsCRP level was obtained by nephelometric assay (Behring Nephelometer Analyzer, Marburg, Germany).

### Statistical analysis

Data distribution was examined by Kolmogorov-Smirnov test. Normally distributed data were presented as arithmetic mean±standard deviation and compared by one-way analysis of variance with Tuckey *post hoc* test. Data with distribution that achieved normality after logarithmic transformation were presented as geometric mean and 95% Confidence Interval and compared by one-way analysis of variance with Tuckey *post hoc* test. Skewed distributed data were presented as median (interquartile range) and compared by Kruskal-Wallis and Mann-Whitney tests, depended on the number of tested groups. Categorical variables were compared by Chi-square test for contingency and presented as absolute frequencies.

Associations of endocan and anthropometric data were tested by non-parametric Spearman's correlation analysis and data were presented as Spearman's coefficient correlation (ρ). Ordinal regression analysis was used to estimate potential independent prediction of anthropometric markers (independent variables) on endocan levels (dependent variable given as tertiles). All the assumptions for univariate and multivariate ordinal regression analysis were taken into account when performing such statistical analysis. Those were as follows: model fitting information had significant Chi-square value (*P*<0.05), goodness-of-fit test had *P*>0.05 and test of parallel lines had *P*>0.05. Also, anthropometric markers were tested for multicollinearity and those which were in mutual collinearity (VIF>4) were excluded from the models. Inclusion criteria for markers entering the models were those that had significant correlation with endocan levels when tested by Spearman's correlation analysis for continuous variables and statistical significant difference between endocan tertile groups tested by Chi-square test for categorical data. Data from these analyses were presented as the estimated odds ratio (95% Confidence Intervals). The explained variation in endocan levels was given by Nagelkerke R^2^ value. Statistical analyses were performed using SPSS® Statistic version 22 (Chicago, USA). Two-tailed *P* values less than 0.05 were set as statistically significant.

## Results

Anthropometric characteristics of study population according to endocan tertiles levels were shown in Table 1. Tested groups had similar gender, hypolipidemic and antihypertensive users distribution. Also, examinees were of similar age in all three groups. The lowest number of smokers was in the third endocan tertile group. The patients in the highest endocan tertile group had higher weight, BMI, WC, HC, WHtR, BAI, BRI and LAP than those from the first and the second tertile group.

**Table 1:** Anthropometric data according to endocan tertiles Data are presented as arithmetic mean ± standard deviation and compared by one-way ANOVA with Tuckey *post hoc* test. ^†^Data are presented as median (interquartile range) and compared by Kruskal-Wallis and Mann-Whitney tests. ^††^ Data are presented as geometric mean (95% Confidence Interval) and compared by one-way ANOVA with Tuckey *post hoc* testafter logarithmic transformation. a-significantly different from the first endocan tertile group b-significantly different from the second endocan tertile group. *P<0.001; # *P*<0.01, ‡ *P*<0.05. BMI-Body mass index; WC-Waist circumference; HC-Hip circumference; WHtR-Waist-to-Height ratio; WHR-Waist-to-Hip ratio; BAI-Body adiposity index; CI-Conicity index; BRI-Body roundness index; ABSI-A body shape index; CMI-Cardiometabolic index; VAI-Visceral adiposity index; LAP-Lipid accumulation product index.

	The first endocan tertile group	The second endocan tertile group	The third endocan tertile group	*P*
N (male/female)	59 (21/38)	59 (15/44)	59 (16/43)	0.430
Age, years	58.9±10.4	60.9±9.2	61.8±9.5	0.270
Smokers, (yes/no)	18/41	15/44	5/54	0.009
Hypolipidemics, (yes/no)	15/44	19/40	12/47	0.337
Antihypertensives, (yes/no)	25/34	33/26	35/24	0.149
Weight, kg^†^	77 (70–88)	76 (66–88)	81 (76–94)^a‡,b#^	0.007
Height, cm^†^	170 (164–175)	168 (162–172)	165 (162–174)	0.282
BMI, kg/m^2†^	26.8 (25.2–28.6)	26.8 (24.0–29.0)	30.5 (27.9–32.6)^a*,b* ^	<0.001
WC, cm	95±9	94±11	100±9^a‡,b#^	0.001
HC, cm	105±7	105±9	111±7^a*,b*^	<0.001
WHtR^†^	0.55 (0.53–0.58)	0.55 (0.51–0.60)	0.59 (0.56–0.63)^a*,b*^	<0.001
WHR	0.90±0.07	0.89±0.07	0.90±0.06	0.384
BAI	29.51±4.63	30.53±4.68	33.66±4.59^a*,b# ^	<0.001
CI	1.28±0.07	1.27±0.08	1.29±0.06	0.426
BRI^††^	4.42 (4.18–4.68)	4.37 (4.06–4.71)	5.31 (5.04–5.60)^a*,b* ^	<0.001
ABSI	0.08±0.01	0.08±0.01	0.08±0.01	0.827
CMI^†^	0.46 (0.35–0.99)	0.75 (0.37–1.11)	0.96 (0.50–1.26)	0.058
VAI^††^	15.1 (12.1–18.8)	1.73 (1.46–2.06)	1.91 (1.60–2.27)	0.128
LAP^†^	45.98 (31.51–73.01)	59.40(35.25–84.09)	78.00^ a*,b*^(49.66–99.90)	0.006
Endocan, ng/L	1.51 (1.21–1.88)	30.3 (24.5–34.9)^a*^	84.9 (59.3 –105.4)^a*,b*^	<0.001

Non-parametric Spearman's correlation analysis revealed that endocan correlated positively with age, weight, BMI, WC, HC, WHtR, BAI, BRI, CMI, VAI and LAP (Table 2). However, the mentioned correlations are relatively weak.

**Table 2:** Spearman’s correlation analysis of endocan and anthropometric characteristics Data are presented as correlation coefficient Rho (ρ). BMI-Body mass index; WC-Waist circumference; HC-Hip circumference; WHtR-Waist-to-Height ratio; WHR-Waist-to-Hip ratio; BAI-Body adiposity index; CI-Conicity index; BRI-Body roundness index; ABSI-A body shape index; CMI-Cardiometabolic index; VAI-Visceral adiposity index; LAP-Lipid accumulation product index.

	Rho (ρ)	* P *
Age, years	0.180	0.017
Weight, kg	0.154	0.040
Height, cm	-0.110	0.145
BMI, kg/m^2^	0.284	<0.001
WC, cm	0.215	0.004
HC, cm	0.342	<0.001
WHtR	0.303	<0.001
WHR	-0.030	0.695
BAI	0.333	<0.001
CI	0.078	0.304
BRI	0.299	<0.001
ABSI	-0.033	0.664
CMI	0.175	0.019
VAI	0.149	0.042
LAP	0.227	0.002

Anthropometric data that showed significant Spearman's correlation coefficient were further tested by regression analysis. Results of univariate and multivariate ordinal regression analysis of endocan tertile levels as dependent variable and anthropometric data as independent variables were given in Table 3.

**Table 3:** Estimated odds ratios after ordinal regression analysis for endocan tertile levels Model included age, BMI, BAI, BRI, CMI, LAP (all continuous variables) and smoking status (categorical variable). BMI – Body mass index; WC – Waist circumference; HC – Hip circumference; BAI – Body adiposity index; BRI – Body roundness index; CMI – Cardiometabolic index; VAI – Visceral adiposity index; LAP – Lipid accumulation product index

	Unadjusted		
	OR (95% CI)	*P*	R^2^
Age, years	1.024 (0.995–1.053)	0.101	0.017
Weight, kg	1.019 (1.001–1.039)	0.043	0.027
BMI, kg/m^2 ^	1.141 (1.062–1.225)	<0.001	0.090
WC, cm	1.038 (1.010–1.066)	0.008	0.048
HC, cm	1.082 (1.043–1.124)	<0.001	0.118
BAI	1.151 (1.082–1.225)	<0.001	0.133
BRI	1.559 (1.230–1.978)	<0.001	0.090
CMI	1.835 (1.106–3.043)	0.019	0.040
VAI	1.215 (1.000–1.475)	0.050	0.026
LAP	1.012 (1.003–1.020)	0.005	0.050
	** Adjusted – Model **		
	** OR (95% CI) **	***P***	**R^2^**
BAI	1.120 (1.036–1.212)	0.004	0.201
BRI	1.183 (0.745–1.876)	0.477	
CMI	2.599 (1.006–6.712)	0.049	
LAP	0.990 (0.973–1.008)	0.269	

Univariate regression analysis demonstrated significant positive correlations of endocan and almost all anthropometric data. Positive association of endo-can and VAI had borderline statistical significance. To explore the independent associations of endocan and anthropometric data, the Model which fulfilled criteria for ordinal regression testing was created. Adjusted odds for BAI given in the Model (OR=1.120, 95% CI 1.036-1.212, *P*=0.004), demonstrated that a rise in BAI by 1 unit increased the probability of higher endocan concentration by 12%. As well, adjusted odds for CMI reached statistical significance, demonstrating that a rise in CMI for 1 unit, raised probability for higher endocan levels for 2.6 times (OR=2.599, 95% CI 1.006-6.712, *P*=0.049). Nagelkerke R^2^ for the Model was 0.201 which indicated that 20.1% of variation in endocan levels could be explained by the Model.

## Discussion

As far as we are aware, this is the first study that evaluated potential relationship between endocan and non-traditional anthropometric indices.

The results of the current research show that not only that serum endocan levels positively correlated with the majority of examined anthropometric parameters in univariate regression analysis, but novel obesity indexes, such as BAI and CMI correlated with this biomarker independently in adult population.

Similarly, the utility of BAI and CMI for estimating the cardiometabolic risk factors, such as the risk of hypertension and hyperuricemia [19] [27] was also shown in previous studies.

Our findings are opposite to previous reports of the inverse association between endocan level and traditional obesity indexes, BMI and/or WC [12] [13] [14] [15]. The possible discrepancies in such results may be explained with the age and sex differences, as well as different ethnic groups that previous studies included. Also, the extent of obesity and phenotypes of adiposity vary significantly among individuals which may also explain this controversies.

The BMI is the most common used parameter for the general obesity assessment. However, it has some drawbacks, which can not give information on the body shape and distribution of adipose tissue, neither can distiguish fat depots and muscle mass [19].

The WC, WHR, and WHtR can provide better information on abdominal obesity than BMI. But the latter ones are limitted to fully differentiate visceral from subcutaneous fat [19].

In order to overcome these drawbacks of traditional anthropometric parameters, some novel obesity indices are emerged [27].

The BAI is derived from HC and height measurements and estimates subcutaneous adipose tissue and the body fat percentage better than BMI [20], and represents better screening tool for coronary risk in adult population than BMI [28]. The three other indices that assess body shape, BRI (i.e. derived from height and WC), CI and ABSI (i.e. both calculated from weight, height and WC) are also shown to be convenient predictors of body fat distribution [21] [22] [23].

The new indicators of abdominal obesity (i.e. CMI, VAI, and LAP), beside WC include lipid parameters to better differentiate subcutaneous from visceral adipose tissue, and to better discriminate cardiometabolic risk than traditional indices [24] [25] [26]. Additionally, CMI besides WHtR, include TG/HDL-c ratio [24] which inversely correlates with the plasma level of small dense LDL particles, that are more atherogenic than larger LDL-particles [29].

In our study, among all examined traditional and novel anthropometric indexes, BAI and CMI correlated independently with endocan in adult population. These findings might enable easier assessment of phenotypes of adiposity that are related to higher endocan level, and consequently higher cardiometabolic risk. These indexes are cost-effective and easy-obtained in everyday clinical practice.

The first study that examined endocan in relation to adipose tissue was conducted by Wellner et al. [30] showing that adipocytes express endocan (previously known as endothelial specific molecule, ESM-1). In line with this Janke and al. [11] reported lower serum endocan levels in overweight/obese postmenopausal women. They also provided subcutaneous abdominal adipose tissue from those women and showed higher expression of endocan in human adipocytes than in preadipocytes. Interestingly, after weight loss programme, serum endocan levels significantly increased, but its expression in adipocytes did not change, suggesting that some other sites of endocan secretion may be more important (e.g. lungs, kidneys) [11].

Our study included more participants that Janke's et al. [11] did and examined both genders. This might explain discordant results. Additionally, we excluded each participant with acute inflammation, to diminish confounding efects on serum endocan level. Namely, it was shown that many pro-inflammatory cytokines induce endocan expression in vitro (e.g., interleukin-1, vascular endothelial growth factor, tumor necrosis factor-alpha, transforming growth factor-beta-1, fibroblast growth factor-2) [31], and some mytogens in adipocytes (e.g., retinoic acid and phorbol ester) [30].

Recently, endocan expression was also reported to be increased in the human placenta from obese women with gestational diabetes mellitus [32] and in reaction to pro-inflammatory stimuli, thus implying that endocan may take part in the states caracterized with increased inflammation. Indeed, not only that the expression of this biomarker is increased by different cytokines' stimuli, but endocan *per se* can trigger endothelial cells to secrete a wide spectrum of other cytokines and enhance leukocytes migration. Moreover, its influence on increased blood vessels permeability is detected, which altogether makes this biomarker reliable for increased cardiometabolic risk recognition [33].

The limitation of this study is its cross-sectional design which can not confirm the causal relationship between endocan and obesity indexes. Furthermore, in the second and the third endocan tertile group there were more females than males than in the first group, and the gender effect might be some source of bias. As well, patients were not asked to discontinue antihypertensive and lipid lowering therapy, which might affect serum endocan levels, as well as some anthropometric indices (CMI, VAI, LAP). At last, we were not able to use precise imaging techniques, for more accurate assessment of visceral compartments. However, contrary to some previous studies, we have shown the positive association of endocan with a wide spectrum of anthropometric indices, both traditional and non-traditional ones. Future studies which will include participants with obesity, without other comorbidities and without any medicament therapy use, might be useful to better evaluate the influence of obesity on serum endocan level.

## Conclusion

Non-traditional obesity indices, BAI and CMI independently correlated with higher serum endocan levels in adult population. Screening for obesity with paying attention on both, total body fat (determined by increased BAI) and visceral adipose tissue distribution (defined by higher CMI) might be useful to differentiate patients with higher endocan levels, and consequently higher cardiometabolic risk. New prospective studies are needed to confirm our results and enlighten the causal relationship between endocan and obesity indexes.

## Acknowledgement

This work was financially supported in part by a grant from the Ministry of Science, Montenegro and the Ministry of Education, Science and Technological Development, Republic of Serbia (project number 451-03-68/2020-14/200161).

## Conflict of interest statement

All the authors declare that they have no conflict of interest in this work.
